# 15-Year-Old Patient with an Unusual Alpha-Fetoprotein-Producing Sertoli-Leydig Cell Tumor of Ovary

**DOI:** 10.1155/2022/4759826

**Published:** 2022-04-12

**Authors:** Kaçar Serife, Stavros Karampelas, Nathalie Hottat, Christine Devalck, Katherina Vanden Houte

**Affiliations:** ^1^Department of Pathology, Brugmann University Hospital Center, Université Libre de Bruxelles, Belgium; ^2^Department of Gynecological Surgery, Brugmann University Hospital Center, Université Libre de Bruxelles, Belgium; ^3^Department of Medical Imaging, Brugmann University Hospital Center, Université Libre de Bruxelles, Belgium; ^4^Pediatric Oncology Reine Fabiola Children's University Hospital, Université Libre de Bruxelles, Belgium

## Abstract

Ovarian Sertoli-Leydig cell tumors (SLCTs) are extremely rare ovarian sex-cord stromal tumors. Alpha-fetoprotein (AFP) production by SLCTs is a rare event generally linked to the presence of hepatocytes or intestinal mucinous epithelium as heterologous elements. We report here a case of a 15-year-old female complaining about abdominal pain, constipation, and spaniomenorrhea with high level of serum AFP leading to a clinical suspicion of malignant germ cell tumor. Final histopathological diagnosis was a moderately differentiated Sertoli-Leydig cell tumor of the ovary with alpha-fetoprotein-producing cells without hepatocytic or intestinal epithelium differentiation. NGS analysis showed mutation in DICER1 gene. SLCTs occur in patients at any age with a mean age of 25 years. The presence of alpha-fetoprotein-producing cells is an important tool in the differential diagnosis of germ cell tumors and challenging in this case of SLCT because of its rarity in this context. An adequate sampling and exhaustive immunohistochemical analyses are mandatory to make the correct differential diagnosis and confirm the presence of alpha-fetoprotein-producing cells and also define the differentiation because of therapeutic strategies between conservative surgery and/or chemotherapy.

## 1. Introduction

Ovarian Sertoli-Leydig cell tumors (SLCTs) are rare mixed-sex-cord stromal tumors of the ovary, concerning less than 0.5% of all primary ovarian neoplasms [1]. It affects all age groups of patients, with 75% detected in young women less than 30 years (mean age of 25 years) and less than 10% detected after menopause. Most of the cases (97%) are unilateral and confined to the ovary at the diagnosis (FIGO stage I) [2].

Due to testicular-like tumor cell types (Sertoli and Leydig cells) producing androgens, clinical signs and symptoms such as irregular menstruation and virilization are seen in 50% of cases with occasional patients presenting estrogenic manifestations [3, 4]. Usually, patients return to normal hormone levels after surgical excision.

Moderately and poorly differentiated forms are the most common types and may contain heterologous elements such as hepatocytes, intestinal type mucinous epithelium, cartilage, and skeletal muscle.

SLCTs are associated with somatic and germline mutations of *DICER1* and *FOXL2* [5].

The overall prognosis is favorable but is related to the degree of differentiation and stage of the tumor [2].

We report here a case of ovarian SLCT in a 15-year-old female with elevated testosterone and alpha-fetoprotein (AFP) without specific differentiation or heterologous elements and review SLCTs producing AFP reported in the literature.

## 2. Material and Methods

Tissue was obtained at the surgery time and fixed in buffered 10% formalin. All sections were stained with routine hematoxylin-eosin saffron.

Immunostainings were performed using a Benchmark Ultra immunostainer (Roche). The panel of antibodies used was Calretinin (SP65 clone, dilution 1/25, Sanbio), Inhibin (Alpha R1 clone, ready to use, Roche), Wilm's Tumor gene (WT1) (6F-H2 clone, ready to use, Roche), Pankeratins (CKAE1/AE3) (PCK26 clone, ready to use, Roche), CD10 (56C6 clone, ready to use, Agilent), EMA (E29 clone, dilution 1/300, VWR), ChromograninA (LK2H10 clone, ready to use, Roche), Synaptophysin (SP11 clone, ready to use, Roche), Hepatocyte Paraffin 1 (HepPar1) (OCH1E5 clone, ready to use, Roche), alpha-1-antitrypsin (Rabbit Polyclonal clone, ready to use, VWR), and alpha-fetoprotein (AFP) (Rabbit Polyclonal clone, ready to use, Agilent). All stainings were controlled with external positive control tissues.

Slides and blocks were sent at Institut Curie (Paris) for complementary studies such as NGS analysis for *DICER1* mutation and OCT3/4, SALL4 immunostainings.

**Figure 1:**
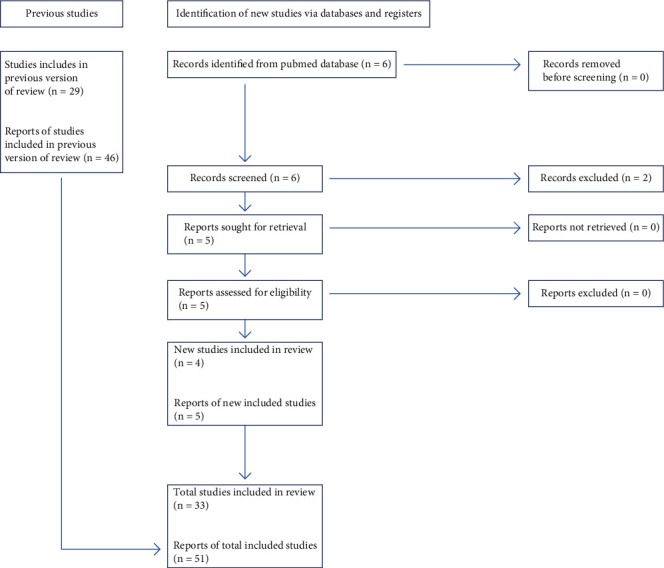
PRISMA 2020 flow diagram for updated systematic reviews [7].

A literature review was made on Pubmed using the keywords: “Sertoli tumor,” “Sertoli-leydig tumor,” and “alpha-foetoprotein.” We searched for cases of SLCTs with elevated AFP levels and associated with specific differentiation and/or heterologous elements or not; in order to understand the origin of AFP production. Only English articles were included. We treated especially the recent largest literature review published by AL-Hussaini et al. made an update with new case reports found on Pubmed using the PRISMA flow diagram for updated systematic reviews [6, 7] (Figure 1).

## 3. Case Presentation

A 15-year-old female presenting complaints of abdominal pain, constipation, and spaniomenorrhea for three months was referred to the department of gynecology. The patient reported menarche at the age of 11 with regular menstrual cycles. No familial history of malignancy was reported. At the physical examination, no abnormalities were noted.

**Figure 2:**
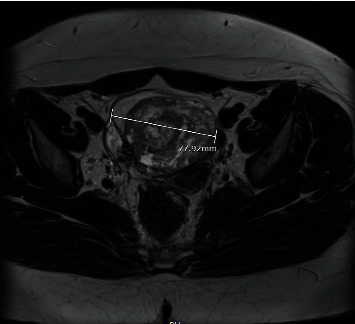
MRI findings. Right adnexal mass with a solid and cystic appearance.

Axial T2-weighted magnetic resonance imaging (MRI) showed a right adnexal mass measuring 7.8 × 6.4 × 7 cm. Left ovary and uterus were unremarkable. There were not any ascites (Figure 2).

Elevated testosterone (66, 5 pmol/L; normal range 3.0-37, 0) and AFP (117 ng/mL; normal range at 14) were noted. Other ovarian tumor markers including beta-human chorionic gonadotropin (*β*-HCG), CA 125, CA 19-9, and carcinoembryonic antigen (CEA) remained negative.

A germ cell tumor (dysgerminoma and yolk sac tumor) or a pure sex cord tumor (juvenile granulosa cell tumor) was clinically suspected.

Laparoscopic right salpingo-oophorectomy was carried out after a multidisciplinary oncological concertation. No adverse event was encountered after surgery.

**Figure 3:**
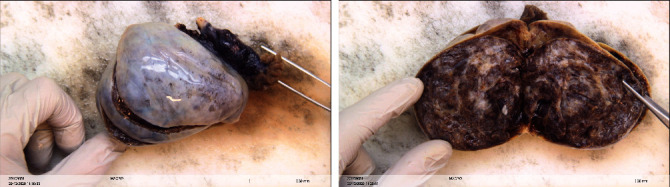
Macroscopic aspect of the tumor. Enlarged ovary replaced by a solid and partially cystic tumor. Solid areas appear yellow-brownish in color with multiple foci of fibrous tissue. Cystic part filled with clear yellow fluid.

Macroscopic examination revealed a right ovary totally replaced by a tumor measuring 8, 5 × 5 × 4, 5 cm with an intact capsule (Figure 3). Gross sections demonstrated a brownish solid aspect with fibrous areas and partially cystic zones filled with clear yellow fluid.

Microscopic examination showed a tumor displaying a multinodular pattern with heterogenous cellularity and an intact capsule.

Cellular zones were composed of Sertoli-like spindle cells having scant-to-moderate amount of cytoplasm with round to oval nuclei and 0 to 1 little nucleoli. At the periphery of these areas, there were clusters of larger cells with a pale foamy cytoplasm and oval nuclei. There were also rare isolated cells with abundant clear cytoplasm.

Tumoral cells showed moderate atypia with a mitotic rate less than 2 mitoses per 10 high-power fields.

**Figure 4:**
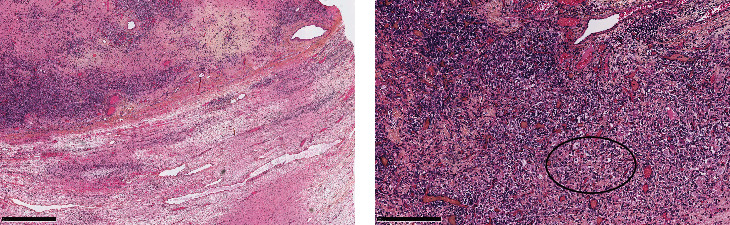
H&E staining at low power view. Cellular lobules are separated by zones of loose fibrous and fibromyxoid mesenchymal stroma (a). Sertoli-like spindle cells with the clusters of Leydig-like cells at the periphery (b).

The stroma containing the tumor was characterized by edema, vascularization, and fibro-sclerotic changes (Figure 4).

**Figure 5:**
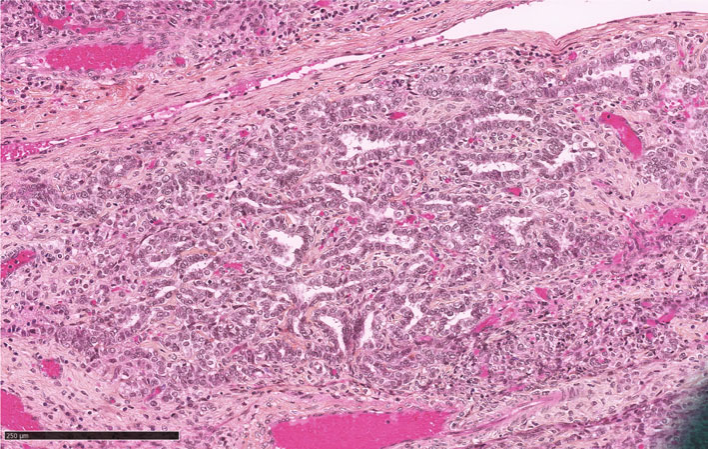
H&E staining at high power view. Area of retiform pattern.

There was one focal zone of retiform pattern less than 1 mm composed of larger cells (Figure 5).

On immunohistochemical studies, Sertoli-like cells showed nuclear staining for WT1, moderately positive cytoplasmic staining for inhibin and weakly positive nuclear and cytoplasmic staining for calretinin.

The Leydig-like cells demonstrated a strong positivity for inhibin and a moderate positivity for cytoplasmic calretinin staining.

**Figure 6:**
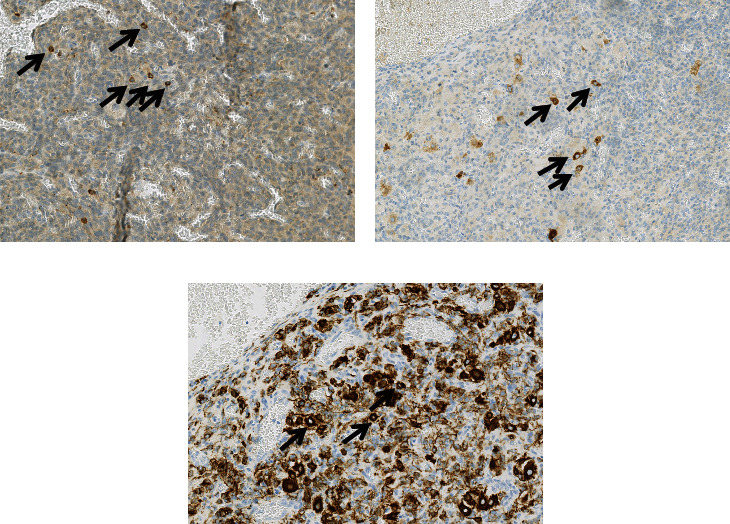
Immunohistochemical studies. Alpha-fetoprotein-producing cells. Positive for and alpha-1-antitrypsin (a), alpha-fetoprotein (b), and cytokeratins (AE1AE3) (c).

Rare isolated cells with abundant clear cytoplasm were positive for cytokeratins (AE1AE3), AFP, and alpha-1-antitrypsin suggesting a hepatocytic differentiation but they did not display any staining for Cytokeratin7 and HepPar1 excluding a complete mature hepatocytic differentiation (Figure 6).

Tumoral cells demonstrated no staining for CD10, EMA, OCT3/4, SALL4, Chromogranin, and Synaptophysin. These stainings excluding the diagnosis of endometrioid carcinoma (CD10 and EMA), embryonal carcinoma (OCT3/4), yolk sac tumor (SALL4), and neuroectodermal-type tumors (ChromograninA and Synaptophysin).

Next-generation sequencing (NGS) revealed somatic DICER1 (c.5113G > A/p. (Glu1705Lys)) and (c.3007C > T/p.(Arg1003∗)) mutations.

Based on the above findings, we concluded to moderately differentiated SLCT with alpha-fetoprotein-producing cells without heterologous elements or specific differentiation (FIGO stage IA). No adjuvant therapy was given.

The patient reported spontaneous menstruation a few days after surgery with improvement of clinical symptoms.

AFP returns to normal range two months after tumor resection. A regular follow-up was planned, and no complaints or clinically relevant abnormalities were noted six months after surgery.

## 4. Discussion

We, hereby, report a rare case of SLCT of moderate differentiation with an unusual AFP-producing cell component and a clinical suspicion of germ cell tumor. In their large literature review, Al-Hussaini et al. reported approximately 50 cases of SLCTs with alpha-fetoprotein-producing component in the last 50 years [6].

We compare our case to these cases and to new case reports since this publication to understand the origin of AFP-producing cells (Table 1) [6, 8–11].

SLCTs present, in 40-60% of the cases, with signs and symptoms of hormone production related to the androgenic activity such as virilization, hirsutism, voice hoarseness, abnormal hair distribution, clitoromegaly, menstrual abnormalities, anovulation, and infertility. These elements are helpful to clinicians to make the differential diagnosis between epithelial and germ cell tumors [4]. SLCTs represent 20% of ovarian sex cord-stromal tumors in children [12].

Laboratory tests are disturbed with elevated plasma testosterone at least 2.5 times its normal value. Elevated serum AFP levels are uncommon in SLCTs, and the significance of this production remains to be elucidated [12]. Tumors that are classically characterized by elevated AFP serum levels include yolk sac tumor, hepatocellular carcinoma, hepatoblastoma, and adenocarcinoma with hepatoid differentiation. Rare reports of female genital tract tumors with serum AFP elevation that concern ovarian neoplasms other than yolk sac tumor are immature teratoma, serous carcinoma, clear cell carcinoma, hepatoid carcinoma, mucinous carcinoma, and carcinosarcoma [6].

In normal situation, AFP is a major fetal plasma component produced in early fetal period by yolk sac, liver, and upper gastrointestinal tract. Its production declines rapidly in a few months after birth and reaches nearly undetectable levels for less than 10 ng/mL [12, 13]. Serum AFP is generally used as tumor marker of hepatocellular carcinomas in adults and germ cell tumors in young patients. Elevated AFP is less frequently identified in lung, oesophagus, stomach, and pancreas carcinomas [14].

The origin of AFP-producing cells in SLCTs remains to elucidate. Several hypotheses have been reported defining AFP-producing cells to be Sertoli-Leydig-like cells, or endodermal derived cells such as hepatocytes or gastrointestinal type mucinous epithelia [15]. Based on the literature review of Al-Hussaini et al., we treated the 29 articles with a total of 46 reported cases.

26 cases presented heterologous elements with AFP production linked to the presence of intestinal-type mucinous epithelium or hepatocytes [15–17]. In their case report, Mooney et al. explained that hepatocytes and Leydig cells have morphological similarity and are closely located to each one [18].

Thus, AFP immunostaining is mandatory to distinguish these cells; Leydig cell is showing a fine cytoplasmic granular positivity for AFP and true hepatocytes displaying a stronger positivity [18].

In the other 20 cases, like our current case with no specific differentiation or heterologous element, the most frequent hypothesis is that AFP-producing cells are Leydig-like cells. The hypothesis is that the presence of crystalloid present in the cytoplasm of Leydig-like cells at HE stain cannot be identified in immunohistochemical stainings because of technical dissolution of crystalloid [6]. Other authors suggest that AFP-producing cells are endodermal sinus differentiation tissue too early to be recognized histologically [12, 19, 20].

Some consider these cells as hepatoid whether or not a clear immunohistochemical staining for HepPar1 is seen [21, 22]. In our case, AFP positive cells displayed immunostaining for CKAE1/AE3 and alpha-1-antitrypsin with no staining for Cytokeratin7 and HepPar1. Moreover, like the article of Ikota et al., the AFP-producing cells were negative for ovarian sex cord-stromal stainings (alpha-inhibin and calretinin). These results support that these cells are neither Sertoli nor Leydig-like cells. Thus, like Ikota et al., we suggest that AFP-producing cells can be derived from an abruptly differentiated tumor element into immature hepatocytes [23].

Talerman and Farley et al. suggest that AFP quantity would be associated to the importance of Leydig-like cell component and tumor size [24, 25]. According to Tiltman et al., retiform pattern that is defined as irregular glandular zone resembling to rete testis seems to be associated with AFP production too [21].

Most authors insist that if AFP staining does not identify any cells, additional tissue sampling is mandatory to identify them [26].

In our complementary literature review, we found four new additional articles reporting five new cases since the last biggest review of Al-Hussaini et al. These cases are associated to heterologous elements and/or hepatocytes and mucinous epithelium with elevated AFP. AFP production is associated to Leydig cells in the case report of Singh et al. or to glandular mucosa cells of the colon in the others [8–11].

According to a large cohort study of Ovarian Sertoli-Leydig cell tumors by Young et al. in 1985, adjuvant chemotherapy is recommended for patients presenting advanced stage, intermediate differentiation, poor differentiation, retiform pattern, and presence of heterologous elements [2]. The optimal treatment algorithm is unknown given the rarity of SCSTs and clinical implications of different AFP producing cell origin are not known.

Furthermore, retiform pattern appears to be a misinterpretation problem and leads to describe it such as endodermal sinus tumor or serous adenocarcinomas [27].

Testing for DICER1 mutations should be performed in all patients with SLCTs because of management and therapeutic consequences. DICER1 mutations can be of germline or somatic type. In the case of germline mutations, called DICER1 syndrome, significant clinical relapse and morbidity occur in patients at young age [28].

## 5. Conclusion

In summary, we reported the case of a 15-year-old female with an unusual type of SLCT and highly elevated serum alpha-fetoprotein levels. AFP and alpha-1-antitrypsin stainings identified isolated AFP-producing cells.

An adequate sampling and microscopic examination are mandatory to identify AFP-producing cells in the cases of SLCT with AFP serum level elevation because of adjuvant therapy management.

These cells are reported to be of several origins such as intestinal-type mucinous epithelium, hepatocytes, Sertoli-Leydig cells, undifferentiated endodermal sinus differentiation tissue, retiform pattern, or abruptly differentiated tumor element into immature hepatocytes. In our case, we consider the AFP-producing cells to be immature hepatocytes.

This case contributes to the available knowledge on the biological, clinical, and histological diversity of SLCTs and specifically at this exceptional form with AFP-producing cells.

## Figures and Tables

**Table 1:** Cases of SLCT with elevated serum AFP: literature review [6, 8–11].

References	Year of publication	No. of cases	Age year	Diagnosis	Heterologous elements	Serum AFP levels before the surgery (ng/mL) (normal range: 10-20) or (IU/mL) 0.0-5.8 IU/ml	Localization of AFP in tumor
Benfield et al.	1982	1	16	Androblastoma	Intestinal-type mucinous epithelium	400	Not performed
Chumas et al.	1984	1	16	Moderately differentiated SLCT	Absent	4 IU/mL	Leydig cells
Young et al.	1984	1	13	Retiform SLCT	Intestinal-type mucinous epithelium and hepatocytes	14 000	Hepatocytes
Sekiya et al.	1985	1	21	SLCT	Absent	109	Unidentified cells
Mann et al.	1986	2	16/16	SLCT	Absent	40/62	Leydig cells/not interpretable
Tetu et al.	1986	1	17	Retiform SLCT	Absent	256	Leydig cells
Tiltman et al.	1986	1	27	SLCT	Absent	153	Leydig cells in the recurrence
Chadha et al.	1987	2	16/11 months	SLCT/retiform SLCT	Intestinal-type mucinous epithelium and hepatocytes	4500/1500	Sertoli cells/hepatocytes
Talerman.	1987	2	Not available	Retiform SLCT	Absent	380-900/7000-11700	Sertoli and Leydig cells
Gagnon et al.	1989	4	17/16/62/18	Retiform SLCT/moderately differentiated SLCT	Absent	256/elevated/not available	Leydig cells
Motoyama et al.	1989	1	18	Retiform SLCT	Intestinal-type mucinous epithelium	1443	Sertoli cells
Taniyama et al.	1989	1	55	SLCT	Absent	105,7	Leydig cells
Larsen et al.	1992	1	55	Bilateral SLCT	Absent	200	Not performed
Farley et al.	1995	1	18	Poorly differentiated SLCT	Absent	850	Not available
Hammad et al.	1995	1	17	Moderately differentiated SLCT	Hepatocytes and carcinoid tumor	194	Hepatocytes
Singh et al.	1996	1	17	SLCT	Intestinal-type mucinous epithelium	40	Leydig cells
Gard et al.	1998	1	17	Moderately differentiated SLCT	Absent	2682	Leydig cells
Mooney et al.	1999	5	44/74/18/23/15	Moderately or poorly differentiated SLCT with retiform pattern	Hepatocytes	Not available but elevated	Leydig cells/hepatocytes
Jang et al.	2002	1	26	Moderately differentiated SLCT	Intestinal-type mucinous epithelium	56,6	Leydig cells
Gheorghisan-Galatenu et al.	2003	1	69	Well-differentiated SLCT	Absent	Not available but elevated	Sertoli cells
Watanabe et al.	2008	1	20	SLCT	Intestinal-type mucinous epithelium	306	Intestinal-type mucinous epithelium
Poli et al.	2009	1	25	Bilateral SLCT	Absent	101 U/mL	Sertoli and Leydig cells
Shu et al.	2012	1	9 months	Moderately differentiated SLCT	Absent	22,02 IU/mL	None
Jashnani et al.	2013	1	22	SLCT	Absent	2925	Leydig cells
Horta et al.	2014	1	19	Poorly differentiated SLCT	Intestinal-type mucinous epithelium	46,3	None interpretable
Liang et al.	2015	1	15	SLCT	Intestinal-type mucinous epithelium	Not available but elevated	Hepatocytes
Lopez-Arias et al.	2015	1	28	SLCT	Hepatocytes	636	Hepatocytes
Ikota et al.	2016	1	12	Moderately differentiated SLCT	Intestinal-type mucinous epithelium	1349,4	Not interpretable
Liggins et al.	2016	1	40	Moderately differentiated SLCT	Hepatocytes and carcinoid tumor	Not available but elevated	Hepatocytes
Al-Hussaini et al.	2017	7	27/20/7/15/18/18/15	Poorly and moderately differentiated SLCT	Intestinal-type mucinous glands	411/100/elevated/137/686/35.5/185	Intestinal-type mucinous epithelium/Leydig cells/none
Xu et al.	2018	2	16/16	Poorly differentiated SLCT	Gastrointestinal mucinous epithelium	919,8/1881	Gastrointestinal mucinous epithelium
Singh C. et al.	2018	1	12	Poorly differentiated SLCT with heterologous rhabdomyosarcoma	Heterologous rhabdomyosarcoma	77,1	Leydig cells
Strus et al.	2019	1	24	Moderately differentiated SLCT with glandular mucosa cells of the colon	Glandular mucosa cells of the colon	10.02 IU/ml	Glandular mucosa cells of the colon
Yamamoto et al.	2019	1	68	Moderately differentiated SLCT	Hepatocytes and hepatocellular carcinomatous tumor cells	Not available	Hepatocytes and hepatocellular carcinomatous tumor cells
Our case		1	15	Moderately differentiated SLCT	Absent	117	Immature hepatocytes

## Data Availability

The data that support the findings of this case report are available from the corresponding author, Kacar Serife.
